# Gallic Acid in the Treatment of Purpura Hœmorrhagica

**Published:** 1853-12

**Authors:** 


In a case of purpura which we had the pleasure of witnessing, in the practice of Prof. James P. White, of this city, the past summer, the exhibition of gallic acid was marked with a gratifying success. Dr. W. prescribed it in the form of a solution in Port wine. It is probably the best hæmostatic yet used in this disease. S. B. H.

### *Gallic Acid in the Treatment of Purpura Hæmorrhagica.*
By T. P. J. Grantham, Esq.

Some years ago I lost one very dear to me from purpura hæmorrhagica; and my attention was thereby strongly directed to that disease, with the hope of discovering some more satisfactory mode of treatment.

The value of gallic acid in passive hæmorrhages induced me to give it a trial in purpura hæmorrhagica, and the results obtained were very gratifying. Moreover, its safety, promptness, and pleasantness, are no inconsiderable recommendations of this remedy.

If the reader should not find any novelty in the application of gallic acid to this disease, the subjoined cases will at least confirm, as far as they go, the value of the treatment.

Case I. Mr. E., a farmer and miller, aged sixty years, came under treatment on the 18th November, 1852. Two days previously, he had noticed a soreness of the tongue, and perceived that the saliva was bloody. The symptoms of purpura were well marked. The gums were spongy, and they bled freely. The tongue and the buccal mucous membrane were dotted with purple fungoid excrescences, some of which were as large as split peas, and from them blood oozed. The breath was offensive, and the appetite was impaired. The urine contained a considerable quantity of blood. Petechiæ were scattered over the thoracic and dorsal regions; and there was a large ecchymosis on one arm, and another on one of the thighs. The gallic acid was administered in five-grain doses every three hours, and two compound rhubarb pills were given at bedtime. On the 20th, the purple excrescences had shrunken and ceased to bleed; the petechiæ and ecchymosis had faded; and the urine was free from blood. On the 30th, the improvement had been sustained. He was quite well again. He said that the first dose of medicine seemed to do him good. Only four scruples were taken.

CASE II. This was a more serious case, several days having elapsed from the first setting in of hæmorrhage from the nose before the patient came under my care.

Master C., aged sixteen years, a draper's apprentice, was seen by me on the 21st June, 1853. He was greatly exhausted and blanched from epistaxis, hæmoptysis, malæna, hæmaturia, petechiæ, and ecchymosis. He was immediately ordered to take gallic acid in doses of three grains every three hours, and subsequently in five-grain doses every two hours. The pil. rhei comp. was given as an aperient. The treatment was followed by most marked benefit, and before a week had elapsed, all hæmorrhage had ceased. On the 5th July, a slight recurrence of epistaxis happened, owing to his having too soon resumed active exercise. But the bleeding was quickly checked by a few doses of the acid, and complete convalescence speedily followed.

CASE III. H. B., aged 12 years, son of an agricultural laborer, after three weeks' illness from typhus, then prevalent in the village where he resided, was seized on the 16th August, 1853, with hæmorrhage from the nose, gums and bowels. The tongue was dotted with purple spots, and the back sprinkled with numerous small petechiæ. The treatment adopted was the same as in the former cases. On the 17th, hæmaturia was superadded to the other symptoms, which were very severe. On the 18th, the hæmorrhages began to abate. On the 20th, the spots on the tongue and the petechiæ had faded; the hæmorrhages had nearly stayed. On the 21st, a severe return of epistaxis occurred, from picking his nose. This was followed by alarming prostration of strength. He was ordered to take the acid every hour, and to have his nose plugged. On the 22d all bleeding had ceased. On the 30th I found that there had been steady and daily improvement since last report. On the 31st he was convalescent.

This was a severe case, as the patient's strength had been so much reduced by a previous exhausting disease; and, accordingly, it was necessary to push the acid to a considerable extent.

In all the cases the dietetic and general management were carefully attended to.—*Association Med. Journal.*

